# Moderation effect of community health on the relationship between racial/ethnic residential segregation and HIV viral suppression in South Carolina: A county-level longitudinal study from 2013 to 2018

**DOI:** 10.3389/fpubh.2022.1013967

**Published:** 2023-01-09

**Authors:** Fanghui Shi, Jiajia Zhang, Xueying Yang, Xiaowen Sun, Zhenlong Li, Chengbo Zeng, Huan Ning, Sharon Weissman, Bankole Olatosi, Xiaoming Li

**Affiliations:** ^1^South Carolina SmartState Center for Healthcare Quality, Arnold School of Public Health, University of South Carolina, Columbia, SC, United States; ^2^Department of Health Promotion, Education, and Behavior, Arnold School of Public Health, University of South Carolina, Columbia, SC, United States; ^3^Big Data Science Center (BDHSC), University of South Carolina, Columbia, SC, United States; ^4^Department of Epidemiology and Biostatistics, Arnold School of Public Health, University of South Carolina, Columbia, SC, United States; ^5^Geoinformation and Big Data Research Lab, Department of Geography, College of Arts and Sciences, University of South Carolina, Columbia, SC, United States; ^6^School of Medicine, University of South Carolina, Columbia, SC, United States; ^7^Department of Health Services, Policy, and Management, Arnold School of Public Health, University of South Carolina, Columbia, SC, United States

**Keywords:** HIV/AIDS, viral suppression, South Carolina, racial/ethnic residential segregation, community health

## Abstract

**Background:**

Viral suppression is the ultimate goal of the HIV treatment cascade and a primary endpoint of antiretroviral therapy. Empirical evidence found racial/ethnic disparities in viral suppression among people living with HIV (PWH), but the evidence of the relationship between racial/ethnic residential segregation and place-based viral suppression is scarce. Further exploring potential structural moderators in this relationship has substantial implications for healthcare policymaking and resource allocation. The current study aimed to investigate the spatial-temporal disparities in the HIV viral suppression rate across 46 counties in South Carolina from 2013 to 2018. We also examined the impact of racial/ethnic residential segregation and the moderation effect of community health, one measurement of community engagement and volunteerism.

**Methods:**

The proportion of PWH who achieved viral suppression for each county and calendar year was calculated using de-identified electronic medical records. The isolation index was calculated and used to measure racial/ethnic residential segregation. The community health index and other county-level factors were directly extracted from multiple publicly available datasets. We used geospatial mapping to explore the spatial-temporal variations of HIV viral suppression rates. Hierarchical quasi-binominal regression models were used to examine the impacts of racial/ethnic residential segregation on county-level viral suppression rate by the extent of community health.

**Results:**

From 2013 to 2018, the average viral suppression rate across 46 counties in SC increased from 64.3% to 65.4%. Regression results revealed that counties with high racial/ethnic residential segregation were more likely to have a low viral suppression rate (β = −0.56, 95% CI: −0.75 to −0.37). In counties with high levels of community health, the impact of racial/ethnic residential segregation on viral suppression rate decreased as compared with those with low levels of community health (β = 5.50, 95% CI: 0.95–10.05).

**Conclusions:**

Racial/ethnic residential segregation acts as a structural barrier to placed-based viral suppression rates and compromises the goal of the HIV treatment cascade. Concentrated and sustained county-level interventions aiming to improve community health can be practical approaches to promote health equity in HIV treatment and care.

## 1. Introduction

Achieving HIV viral suppression can help improve the immune recovery of people living with HIV (PWH) and prevent onward transmission to others, making viral suppression the goal of the HIV treatment cascade (1, 2). In 2019, among the 17,589 PWH in South Carolina (SC), only 70.7% of them achieved viral suppression, and the viral suppression rate among PWH differed by racial/ethnic groups and geographic locations (1, 3–6). Studies investigating the social and structural determinants of viral suppression and identifying the vulnerable communities could facilitate health policymaking and community intervention development, which are critical for promoting health equity in HIV treatment and care.

Based on the HIV care cascade model, there is a cascade relationship between three crucial steps of the HIV care continuum, including linkage to care, retention in care, and viral suppression (7). According to the Joint United Nations Program on HIV/AIDS (UNAIDS), 95% of all PWH should know their HIV status, 95% of people with diagnosed HIV receive sustained antiretroviral therapy (ART), and 95% of all PWH receiving ART should achieve viral suppression by the year 2025. The structural racism, which refers to macro-level systems, institutions, ideologies, social forces, and processes that generate and reinforce inequities among racial/ethnic groups, could be salient population-level determinants of the HIV care continuum outcomes (8). Residential segregation, the geographic separation of racial groups' homes, is one pervasive type of structural racism, and it constitutes a significant roadblock to the 2030 95–95–95 targets launched by UNAIDS (9–11).

Racial/ethnic residential segregation leads to health disparities through unequal access to socioeconomic opportunities, neighborhood environment, and health resources in segregated areas (9, 11, 12). Historical structural racism in housing policies impeded racial/ethnic minorities in the US from owning property and building wealth, resulting in racial/ethnic minorities disproportionately living in disadvantaged communities with fewer resources and a more hazardous environment (13). In the case of HIV viral suppression, limited access to health care services in resource-restricted communities due to residential segregation may lead to suboptimal viral suppression by disrupting the linkage and retention in the HIV care continuum cascade (14, 15).

Examining the moderation effect outside the individual level can provide vulnerable insight into the complex relationship between racial residential segregation and viral suppression (13). For example, it is possible that the impact of segregation on viral suppression varied according to the level of community participation and engagement. Uncovering the differential effect of segregation by neighborhood characteristics enables tailored public health interventions and strategies to improve viral suppression. Adequate community participation and engagement could mitigate the negative impact of racial/ethnic residential segregation on viral suppression (16). Based on the “HIV-competent community” framework, people with more participation and engagement in community activities are more likely to respond collaboratively and effectively to HIV treatment and care (17). In racially segregated regions, a high level of community participation and engagement (refers to better community health) could promote social cohesion and social support, facilitating access to better socioeconomic opportunities, neighborhood environment, and health resources (17). PWH living in racially segregated communities with higher levels of participation and engagement are more likely to be linked to care and retained in HIV treatment, increasing the likelihood of viral suppression. However, research evidence regarding the protective effect of community health is limited.

In this study, we conducted area-based research to describe the spatial-temporal trends of viral suppression rate among PWH across the 46 counties in SC from 2013 to 2018. Additionally, we examined the longitudinal impact of racial/ethnic residential segregation on county-level viral suppression by the different extent of community participation and engagement.

## 2. Methods

### 2.1. Participants

All adult PWH (>18 years of age) diagnosed with HIV in SC from January 2013 to December 2018 were included in this study. Their de-identified laboratory data were extracted from the electronic HIV/AIDS reporting system (eHARS) in the SC Department of Health and Environment Control (DHEC) (18).

### 2.2. Measurements

#### 2.2.1. The county-level viral suppression rate

Using the eHARS data, the annual county-level viral suppression rate was calculated as the percentage of PWH with a viral load of fewer than 200 copies per ml in each county's last viral load report at each calendar year (excluding those newly diagnosed in that year) (19). Individuals without viral load records in the calendar year (ranging from 30.78% to 33.48%) were excluded from the analysis in that year. Still, they were included in the analysis for other calendar years. This calculation criterion is in line with the US Department of Health and Human Service's calculation (20).

#### 2.2.2. Racial/ethnic residential segregation

We calculated the racial/ethnic residential segregation using Massy and Denton's formula of isolation index for non-Hispanic black residents (21). The isolation index is suggested to measure racial/ethnic residential segregation regarding infectious disease as it reflects the probability that a minority person shares a unit area with another minority person (22). In this study, the non-Hispanic black isolation index for a county is calculated as follows:


$$\begin{array}{l} \sum_{i=1}^{n}[\left(\frac{\text{Total number of non}\_\text{Hispanic black residents in census tract }}{\text{Total number of non}\_\text{Hispanic black residents in the county}}\right)\\ \;\left(\frac{\text{Total number of non}\_\text{Hispanic black residents in census tract}}{\text{Total population in the census tract}}\right)] \end{array}$$

In this calculation, *i* is the ith census tract in the county, and *n* is the number of census tracts in the county. The isolation index reflects the probability that non-Hispanic black residents will come across others of the same race/ethnicity, and the index ranges from 0.0 (complete integration) to 1.0 (complete segregation) (23).

#### 2.2.3. Community health

We extracted the community health index for each county directly from the US Congress Joint Economic Committee website (16). Community health was calculated based on the registered non-religious non-profits per 1,000, religious congregations per 1,000, and Informal Civil Society Sub-Index (constructed from various state-level sources, such as the share who volunteered, who attended public meetings, and who participated in political activities) (16). The original values of these indicators were standardized, weighted based on principle components analysis, and summed to generate the community health index. The value of community health for the 46 counties in South Carolina ranges from −1.09 to 4.12, with a higher score indicating a higher level of community engagement and volunteerism in the local area.

#### 2.2.4. Confounders

Based on existing literature on the social and structural determinants of HIV viral suppression (24), we summarized the potential confounders into three categories: (1) population composition (e.g., percent of male, percent of the population who were at least 18 years old); (2) socioeconomic characteristics (e.g., percent of persons with income below poverty level, percent of the population who were unemployed); and (3) healthcare access (e.g., percent of persons with no health insurance coverage, the number of Ryan White HIV centers per 100,000 population within 25 miles radius). We extracted these potential confounders from multiple publicly available datasets, such as the 2014–2018 5-year estimated America Community Survey and the US Congress Joint Economic Committee. The detailed definition and data source of each covariate are displayed in Table 1.

**Table 1:** The detailed description of each variable and data source.

**Variable**	**Variable detail**	**Data source**
Isolation index	The extent to which minority members are exposed only to one another	Calculated using data extracted from America Community Survey 5-year estimates
Community health	Registered non-religious nonprofits per 1,000, religious congregations per 1,000, and informal social activities subindex	The United States Congress Joint Economic Committee website
Male (%)	Percentage of male population	America Community Survey 5-year estimates
Age (≥18, %)	Percentage of persons aged > 17	America Community Survey 5-year estimates
Poverty (%)	Percentage of persons below US poverty level	America Community Survey 5-year estimates
Unemployment (%)	Percentage unemployed	America Community Survey 5-year estimates
No insurance (%)	Percentage of persons under age 65 without health insurance	America Community Survey 5-year estimates
Ryan White HIV centers per 100,000	The number of Ryan White HIV centers within 25 miles radius of each county per 100,000 population	Calculated using information about the geolocation of Ryan White HIV healthcare center in a county extracted from US department of Health and Human Services (DHHHS) data warehouse and the total population in a county extracted from America Community Survey 5-year estimates

We linked the viral suppression rate, residential segregation index, community health index, and potential confounders by the unique Federal Information Processing Standards (FIPS) code of each county.

### 2.3. Statistical analysis

First, we described all continuous variables using three quantities (25th percentile, 50th percentile, and 75th percentile) and interquartile range (IQR). Second, geospatial mapping was employed to describe the spatial-temporal variations of county-level viral suppression rates across 46 counties in SC from 2013 to 2018. Third, we used three quasi-binomial generalized linear mixed models to test the impact of racial/ethnic residential segregation on county-level viral suppression rate by the extent of community health. Model 1 only included the main effects of racial/ethnic residential segregation and community health. In model 2, the main effects and the interaction between racial/ethnic residential segregation and community health were included and examined. All potential confounders (e.g., population composition, socioeconomic characteristics, and healthcare resources) were controlled in model 3. We adjusted for the cluster effect, repeated measures, and time effect in all three models. Quasi-binomial generalized linear mixed models were applied in the current study since the dependent variable is the proportion of PWH with viral load <200 copies/ml and all variables were county-level longitudinal data. Compared to binomial regression, quasi-binomial can fit proportional data without specifying the numerator and denominator (25). Fourth, a Johnson-Neyman interaction plot was generated to illustrate the moderation effect of community health in the relationship between residential segregation and viral suppression. Compared to the pick-a-point technique (one standardized deviation above the mean, mean, and one standardized deviation below the mean), the Johnson-Neyman technology can provide more comprehensive information on how the effect of residential segregation's influence on viral suppression is conditional on the entire range of community health (26). All analyses were conducted in R version 4.1.2. The Institutional Review Boards approved the study proposal at both University of South Carolina and SC DHEC.

## 3. Results

### 3.1. Descriptive statistics

**Figure 1:**
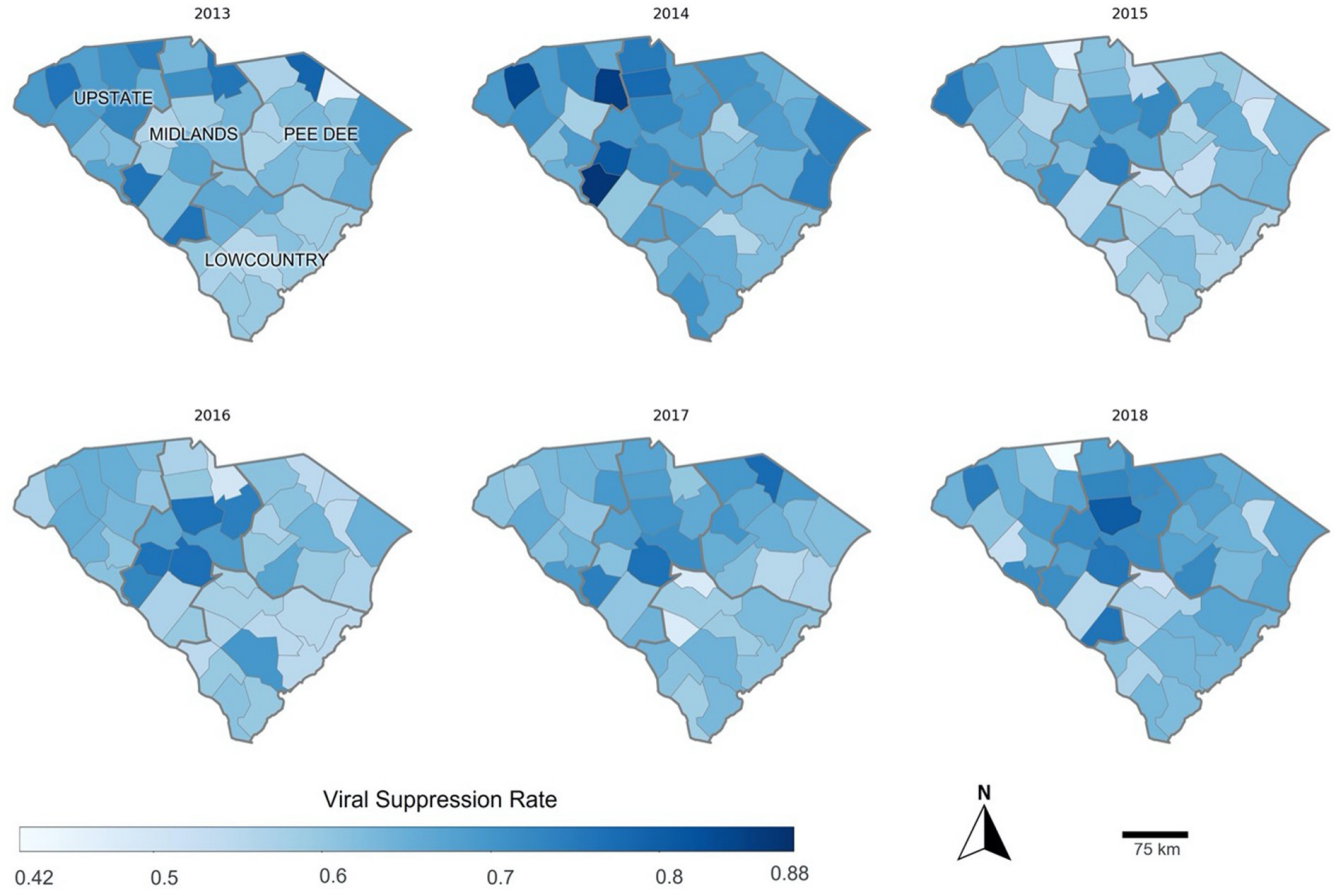
The viral suppression rate across 46 counties in South Carolina from 2013 to 2018.

The median viral suppression rate across the counties from 2013 to 2018 was 64% (IQR: 60%−69%) (Table 2). The geographic variations of viral suppression rates from 2013 to 2018 are demonstrated in Figure 1. Counties with low viral suppression rates are primarily in the Lowcountry and Pee Dee areas. For example, Calhoun County consistently had low viral suppression rates from 2013 to 2018. The median value of racial/ethnic residential segregation was 0.45 (IQR: 0.38–0.59), which reflects that in half of these counties, on average non-Hispanic Black lived in a census tract where nearly half of the residents were also non-Hispanic Black. The median value for community health was −0.55, which is much lower than the national median level, which is −0.23. Across the counties, around half of the residents were male, and more than 75% of the population were at least 18 years old. The variation (IQRs) of poverty, unemployed, and no insurance was lower than 7% but higher than 4%. In more than half of the counties, there was at least one Ryan White HIV center per 100,000.

**Table 2:** Descriptive statistics of county-level viral suppression rate and structural independent variables among 46 counties in South Carolina, 2013–2018.

	**25th percentile**	**Median**	**75th percentile**	**IQR**
Viral suppression rate	0.6	0.64	0.69	0.09
Racial/ethnic residential segregation	0.38	0.45	0.59	0.21
Community health	−0.79	−0.55	−0.31	0.48
Male (%)	48.02	48.56	49.28	1.26
Age (≥18, %)	76.34	77.55	79.22	2.88
Poverty (%)	16	19.36	22.11	6.11
Unemployed (%)	8.05	10.16	12.52	4.47
No insurance (%)	12.06	14.22	16.5	4.44
Ryan White HIV centers per 100,000	0.34	1.6	4.54	4.2

IQR, interquartile range.

### 3.2. The impact of racial/ethnic residential segregation on county-level viral suppression rate by the extent of community health

**Figure 2:**
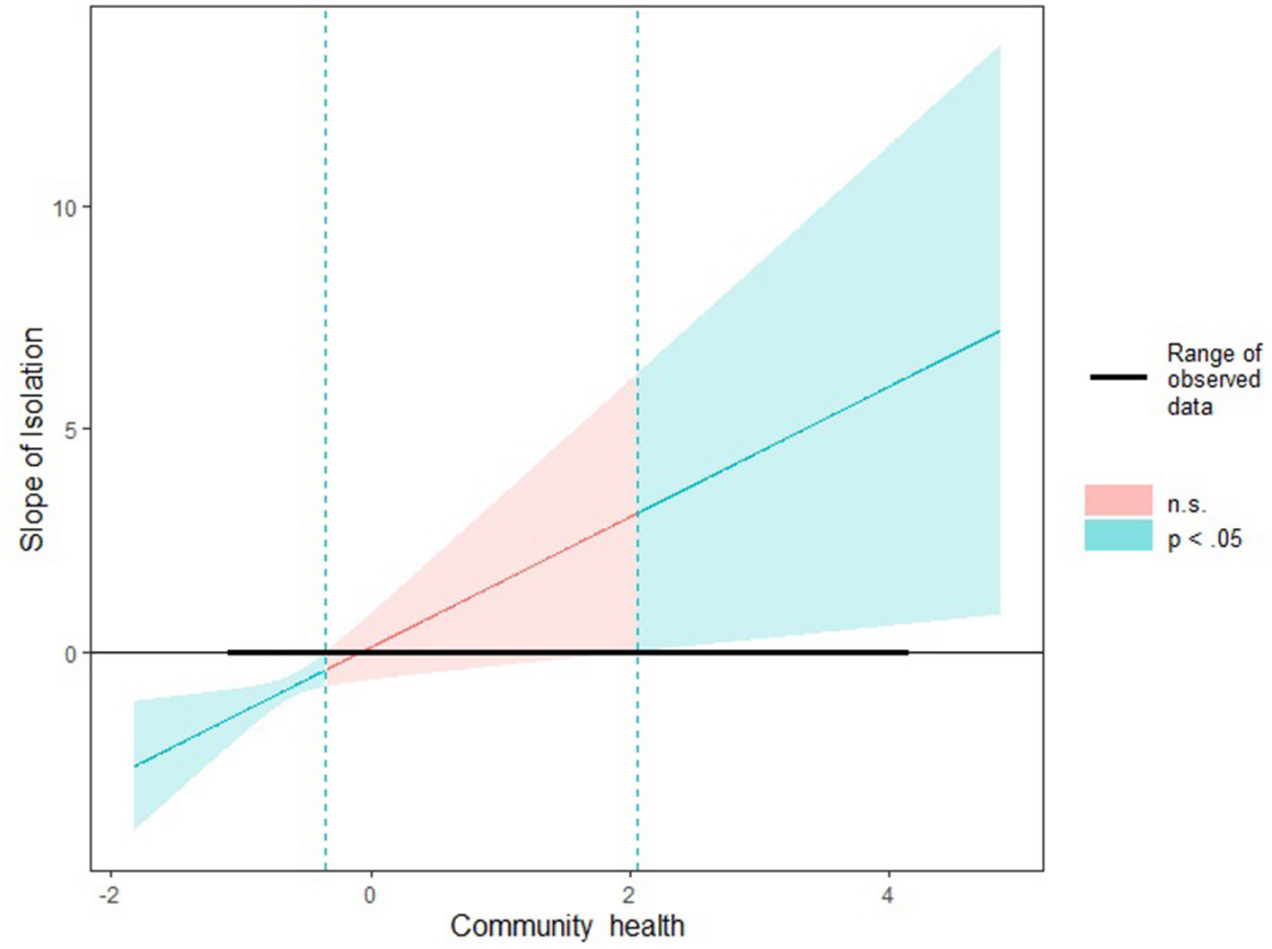
The moderation effect of community health in the relationship between residential segregation and viral suppression: A Johnson-Neyman interaction plot.

In the hierarchical quasi-binomial generalized linear mixed modeling (Table 3), model 1 showed that racial/ethnic residential segregation (β = −0.56, 95% CI: −0.75 to −0.37) was negatively associated with viral suppression rate. In contrast, the community health showed a positive impact (β = 0.59, 95% CI: 0.16 to 1.03). Model 2 showed that the interaction effect between racial/ethnic residential segregation and community health was statistically significant (β = 5.73, 95% CI: 0.36 to 11.09). In model 3, after controlling for potential confounders, the significant interaction term of racial/ethnic residential segregation and community health remained in the model (β = 5.50, 95% CI: 0.95 to 10.05). According to Figure 2, when community health was lower than −0.34, the lower the score of community health, the stronger the negative relationship between the isolation index and viral suppression rate. We didn't explain the moderation effect of community health when it is higher than 2.06 (the upper limit of the Johnson-Neyman range) because there is only one county with a community health value larger than 2.06 among the 46 counties. The significant interaction revealed that the negative influence of racial/ethnic residential segregation on viral suppression among PWH decreased in counties with high levels of community health compared to those with low levels of community health (Figure 2).

**Table 3:** Regression coefficients for viral suppression rate among people living with HIV across 46 counties in South Carolina from 2013 to 2018.

**Predictors**	**Model 1**	**Model 2**	**Model 3**
	**(**β**, 95% CI)**	**(**β**, 95% CI)**	**(**β**, 95% CI)**
Calendar year	0.00 (−0.02 to 0.01)	−0.01 (−0.03 to 0.01)	0.00 (−0.02 to 0.02)
Isolation index (I)	−0.56 (−0.75 to −0.37)^**^	−1.03 (−1.62 to −0.44)^*^	−1.02 (−1.48 to −0.56)^***^
Community health (C)	0.59 (0.16 to 1.03)^*^	−2.29 (−5.03 to 0.45)	−2.06 (−4.36 to 0.24)
I^*^C		5.73 (0.36 to 11.09)^***^	5.50 (0.95 to 10.05)^*^
**Population composition**			
Male (%)			0.02 (−0.17 to 0.21)
Age (≥18, %)			−0.02 (−0.21 to 0.17)
**Socioeconomic characteristics**			
Poverty (%)			0.09 (−0.12 to 0.30)
Unemployed (%)			−0.09 (−0.34 to 0.16)
**Health care access**			
No insurance (%)			0.13 (−0.08 to 0.34)
Ryan White HIV centers per 100,000			−0.23 (−0.53 to 0.07)

* *p* < 0.05,

** *p* < 0.01,

*** *p* < 0.001.

The median (InterQuartile Range) of the deviance residuals are −0.332 (−3.1325, 3.3246) for model 1, −0.0630 (−3.3504, 3.5275) for model 2, and −0.0677 (−3.3789, 3.5663) for model 3.

## 4. Discussion

In the current study, we found that racial/ethnic residential segregation had a negative impact on the proportion of PWH who achieved viral suppression across the counties in SC, and such impact differed by the level of regional community health.

Even though no comparable data are reported in the existing literature, some empirical evidence can help explain the mechanisms through which elevated racial/ethnic residential segregation affect the HIV viral suppression rate, such as concentrated economic inequality, poor community environment, and limited access to resources (8, 11, 22, 25). For example, racial/ethnic disparities in HIV viral suppression can be enlarged due to segregated sexual networks and reduced healthcare resources in areas with a concentrated Black population (8). Shacham et al. found that individuals in residentially segregated neighborhoods were more likely to have difficulty accessing medical care and a higher rate of sexually transmitted infections in their sexual networks (25). By enhancing existing evidence, our finding underscores the effectiveness of addressing county-level racial residential segregation as a strategy to improve HIV treatment cascade outcomes.

The negative impact of racial/ethnic residential segregation on the viral suppression rate differed by the community health levels, suggesting that building community capacity, improving community engagement, and strengthening community cohesion could mitigate the negative impact of racial/ethnic residential segregation on HIV viral suppression (27–31). Social network theories suggest that individuals embedded in groups have greater access to social support and are expected to exhibit lower disease risk (32). The network building and close interface during community volunteerism and engagement help PWH talk openly about their HIV status, change their risk behaviors, and cope with the infection/disease (33, 34). For example, discussing HIV status makes them more likely to receive timely treatment and be retained in care, which is necessary for successful viral suppression (33, 34). In addition, in counties with better community health, PWH have an increased sense of responsibility (35) and a sense of belonging (36), which in turn is associated with better engagement in HIV treatment and a higher possibility of viral suppression (29, 37).

High levels of community cohesion and civic engagement are necessary for satisfactory HIV treatment outcomes, especially in residentially segregated areas by race/ethnicity (34, 38). Communities with adequate engagement could provide PWH with various forms of social support (e.g., information exchange and cash loan), which can mitigate resource deficiency's adverse effect on viral suppression in segregated areas (31). Social support can also mitigate the negative influence of residential segregation on viral suppression by enhancing PWH's ability to link to and engage in HIV treatment which could contribute to successful viral suppression (31). In one multivariate longitudinal study, prayer support in the religious setting was related to positive health outcomes among PWH (39, 40).

The current study shows a complex relationship between county-level residential segregation, community health, and viral suppression rate. Residential segregation had a stronger negative impact on viral suppression in counties with lower community health. This finding informs future tailored resource allocation and public health intervention efforts to reduce residential segregation and enhance community health at the county level. To be more specific, more attention is needed to address residential segregation issues for counties ranking low in community health. Additionally, based on the measurement of community health, increasing the number of non-religious non-profits per 1,000, the number of religious congregations per 1,000, and informal civil society activities (e.g., engagement in volunteerism, religious groups, or community-based antiretroviral therapy adherence groups) can be effective strategies for improving viral suppression in racially segregated areas (35, 41).

Some limitations of the current study need to be acknowledged. First, this county-level study investigated population-level factors but may obscure the importance of individual-level risk factors. We need to incorporate individual-level factors in future studies to investigate the interactive influence of structural factors and individual attributes on HIV viral suppression. Second, the causal inference was limited due to the retrospective nature of the current study design. Third, our study only captures and represents adult PWH in SC. Further work should be extended with the inclusion of PWH beyond adults and more locales affected by HIV elsewhere in the US. Fourth, our analysis focused on the county level due to data availability. Future research efforts are needed to examine the relationships in a more granular geographic unit (e.g., zip code level) because there are important variations in residential segregation and community health within each county. Last, Individuals without viral load records were excluded from calculating the viral suppression rate in that year. The missing measurement of viral load might indirectly reflect dropping out of care or other reasons, such as patients moving out of South Carolina. This might lead to our study either overestimating or underestimating the viral suppression rate. Still, our calculation aligns with the national and state criteria, and we believe it could provide essential information for county-level viral suppression rate disparities in SC.

The current study underscores the importance of taking structural factors into account when aiming to achieve optimal HIV treatment outcomes. Suppressing viral load to undetectable levels (<200 copies/ml) is essential for individuals' long-term health and reducing transmission in a community. To effectively combat and curb the HIV epidemic, there is a need for concentrated and sustained county-level interventions designed to improve the community health of the living environment.

## Data availability statement

Individual level data is not publicly available due to provisions in our data use agreements with state agencies/data providers, institutional policy, and ethical requirements. We make access to such data available *via* approved data access requests. Requests to access the datasets should be directed to olatosi@mailbox.sc.edu.

## Ethics statement

The studies involving human participants were reviewed and approved by the Institutional Review Boards at both University of South Carolina and SC DHEC. Written informed consent for participation was not required for this study in accordance with the national legislation and the institutional requirements.

## Author contributions

FS, JZ, and XY conceived of and designed the study. FS, JZ, XY, XS, HN, and ZL participated in the data extraction and interpretation. FS wrote the first draft. FS, JZ, XY, CZ, XS, ZL, SW, BO, and XL provided critical revisions. All authors contributed to the article and approved the submitted version.
